# Polymorphism rs7214723 in CAMKK1: a new genetic variant associated with cardiovascular diseases

**DOI:** 10.1042/BSR20210326

**Published:** 2021-07-06

**Authors:** Sofia Beghi, Francesca Cavaliere, Matteo Manfredini, Sandro Ferrarese, Claudio Corazzari, Cesare Beghi, Annamaria Buschini

**Affiliations:** 1 Department of Chemistry, Life Sciences, and Environmental Sustainability, University of Parma, Parco Area delle Scienze 11A, Parma 43124, Italy; 2 Department of Food and Drug, University of Parma, Parco Area delle Scienze 27A, Parma 43124, Italy; 3 Department of Cardiac Surgery, Circolo Hospital, University of Insubria, Varese, Italy

**Keywords:** calcium signaling pathway, Cardiovascular diseases, genetic variants, single nucleotide polymorphism

## Abstract

Cardiovascular diseases (CVDs) are the leading cause of deaths worldwide. CVDs have a complex etiology due to the several factors underlying its development including environment, lifestyle, and genetics. Given the role of calcium signal transduction in several CVDs, we investigated via PCR-restriction fragment length polymorphism (RFLP) the single nucleotide polymorphism (SNP) rs7214723 within the calcium/calmodulin-dependent kinase kinase 1 (*CAMKK1*) gene coding for the Ca^2+^/calmodulin-dependent protein kinase kinase I. The variant rs7214723 causes E375G substitution within the kinase domain of CAMKK1. A cross-sectional study was conducted on 300 cardiac patients. RFLP-PCR technique was applied, and statistical analysis was performed to evaluate genotypic and allelic frequencies and to identify an association between SNP and risk of developing specific CVD. Genotype and allele frequencies for rs7214723 were statistically different between cardiopathic and several European reference populations. A logistic regression analysis adjusted for gender, age, diabetes, hypertension, BMI and previous history of malignancy was applied on cardiopathic genotypic data and no association was found between rs7214723 polymorphism and risk of developing specific coronary artery disease (CAD) and aortic stenosis (AS). These results suggest the potential role of rs7214723 in CVD susceptibility as a possible genetic biomarker.

## Introduction

Cardiovascular diseases (CVDs) are the leading cause of deaths in industrialized countries affecting increasing number of people every year [1]. CVDs refer to a wide range of conditions affecting heart function and blood vessels, two of the most frequent being coronary artery disease (CAD) and aortic stenosis (AS).

CAD results from progressive stenosis of coronary arteries caused by the atherosclerosis process. Clinical syndromes are caused by an imbalance between oxygen supply and demand, resulting in a myocardial perfusion inadequate to meet metabolic demand (ischemia). Plaque rupture with superimposed thrombosis is responsible for most acute coronary syndromes and different types of myocardial infarction [2]. AS results from complex and non-completely understood pathophysiologic mechanisms, causing thickening, calcification, and/or fusion of the aortic valve leaflets (LV), determining a variable degree of stenosis in the left ventricle outflow tract [2]

Even if the pathophysiology in CVDs is well known and there are multiple approaches to treat patients, it is important to consider that this kind of conditions are complex because multifactorial [1,3]. In addition to lifestyle habits (tobacco usage, unhealthy diet, alcohol abuse, physical inactivity), it is important to stress that CVDs represent a complex trait with multiple genetic and environmental components. Since complex diseases do not follow a clear pattern of Mendelian inheritance, the study of genetic variants in candidate genes is expected to provide insights into the genetic basis of not only for disease predisposition but also for responses to drugs and aging [4–6].

In recent years, different studies have been focusing on genetic association as an approach for assessing the contribution of specific gene variants to disease risk [4,5,7].

The identification of polymorphisms as prognostic and predictive biomarkers in CVDs could be really important for both preventive screenings and monitoring of patients with specific CVDs [4,5]. Among the several genes involved in the physiology and anatomy of the heart, genetic factors involved in the calcium/calmodulin pathway might play a key role in modifying the individual risk of developing CVDs. The calcium signaling pathway in particular plays a significant role in heart and vessels [8–11], being involved in different processes as for example the regulation of the excitation–contraction mechanism [10,12,13].

In fact, it has been shown that Ca^2+^ regulates the rhythmic contractions of the heart via diverse signaling pathways, creating an interconnected network involved different specific functions in different regions of the heart [10,14].

Within the different calcium signaling pathways, calmodulin has a central role: its function is to integrate the calcium signal and to transduce it to other downstream enzymes, such as NOS, PPP3C as well as the proteins of the CAMK subfamily. In a previous review [14] we focused our attention on the role played by genetic variants in these family of proteins in the development of CVDs [15–21]. Here we focus on CAMKK1, a calcium/calmodulin-dependent kinase kinase 1, functionally located upstream to others CAMK kinases involved in the transduction of calcium signaling.

CAMKK1 has been recently assigned a novel role as regulator of the mesenchimal stem cell (MSC) secretome, specifically of the exosome. It was also demonstrated that direct overexpression of CAMKK1 in infarcted cardiac tissue has therapeutic beneficial effects, improving ejection fraction and decreasing infarct size after acute myocardial infarction [22].

CAMKK1 is a transferase of the Ser/Thr protein kinase family and it belongs to the Ca^(2+)^/calmodulin-dependent protein kinase subfamily [23]. This protein is the most upstream element of CaM-kinase cascade, and it is maintained in a dormant state when calcium levels are basal. When calcium levels increase, calmodulin binds four ions of calcium and activates CAMKK1 for the phosphorylation of downstream proteins [23].

The activity of CAMKK1 is important for different processes, including neuronal differentiation, stress resistance in skeletal muscle, mitochondrial morphology, cell proliferation and neurodegenerative disorders [23–25].

It is supposed that alteration in the *CAMKK1* gene sequence might have an impact on the protein structure and its activity. The T to C variation at rs7214723 in CAMKK1 causes the change in the amino acid glutamate into glycine at the position 375 and has been associated with lung cancer [25,26]. Since this amino acid variation is located in the kinase domain, there is the possibility that such a change could decrease the activity of CAMKK1 and inhibit selected downstream protein kinases, causing specific disorders [25,26].

In the present study, we investigated the polymorphism rs7214723 in CAMKK1 for its potential association with CVDs, with the aim of finding new biomarkers related to an increased risk of developing these diseases, highlighting the role of CAMKK1 in CVDs and providing insights in the physiology and molecular biology of CVDs.

## Methods

### Population study

The study protocol (study number 97/2017) was approved by the Ethical Committees of the Università degli Studi dell’Insubria (Varese, Italy). Informed consent was obtained in accordance with the principles outlined in the Declaration of Helsinki.

The study involved 300 cardiopathic subjects requiring cardiac surgery recruited at the Cardiac Surgery of Varese Hospital (Italy). Of the 300 patients, the percentage of non-Italian subjects was below 5%.

**Figure 1:**
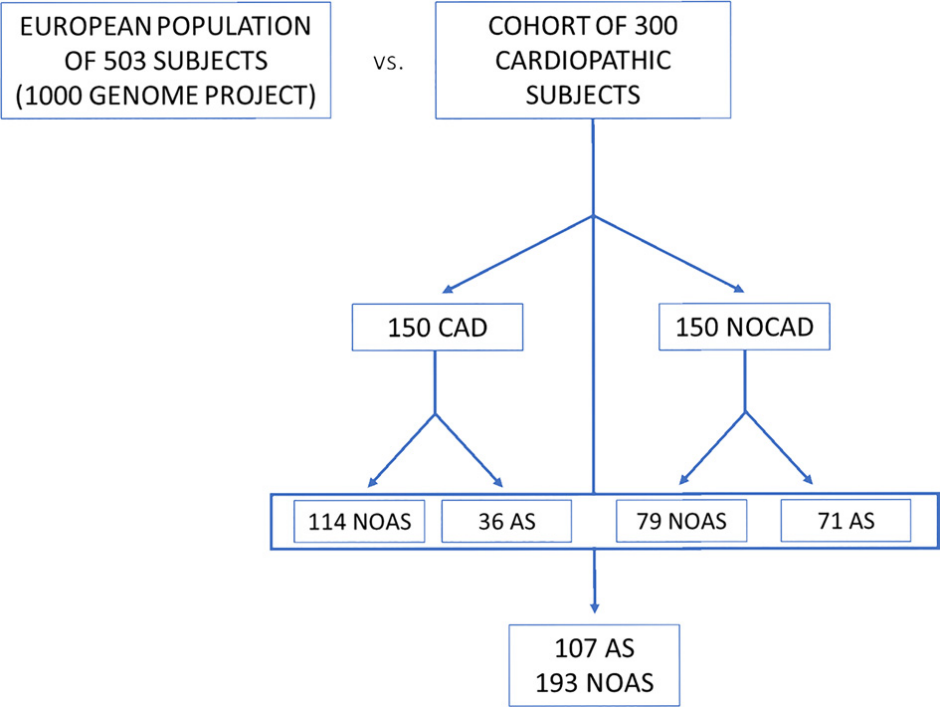
Scheme of the populations object of study European population (reference group) compared with the cardiopathic population. It is also possible to observe how it was designed, the stratification inside the cohort of 300 cardiopathic subjects.

All patients were subjected to clinical (presence/absence of chest pain, comorbidity, and comorbility) and instrumental diagnosis. Patients underwent a series of tests routinely performed before any cardiac operation, including electrocardiogram (ECG), echocardiogram (echo), and coronary angiography. These tests allowed the subdivision of the patients in two subgroups. The first group comprised 150 patients with coronary artery disease (CAD group), while the second included 150 patients without CADs (NOCAD group). Among all the patients, 107 had AS, of which 71 belonged to the NOCAD group (Figure 1).

### Sample collection

Samples were collected from September 2017 to March 2019. Approximately 5 cc of whole blood was collected from all patients via peripheral venous puncture. An aliquot of 125 µl was then spotted on Whatman FTA cards for subsequent genetic analysis.

### DNA extraction and CAMKK1 genotyping

The polymorphism rs7214723 of *CaMKK 1* gene was genotyped by PCR-restriction fragment length polymorphism (RFLP) strategy. DNA was extracted from blood samples spotted on FTA cards following a specific protocol optimized in our laboratory. Briefly, the DNA of each patient was isolated from two 1.2-mm diameter disks of FTA picked up through Harris Micro Punch and subsequently inserted in a PCR tube. Each sample was incubated for 5 min with 200 μl of FTA Purification Reagent. We repeated this step three times, to ensure greater DNA quality for PCR analysis by removing inhibitors and other contaminants. Each sample was then incubated twice for 5 min with 200 μl of TE buffer at pH = 8.0 (Tris-HCl 10 mm pH 8.0; EDTA 1 mm pH 8.0). Each sample was finally left for 10 min at 56°C before being used for the PCR.

**Figure 2:**
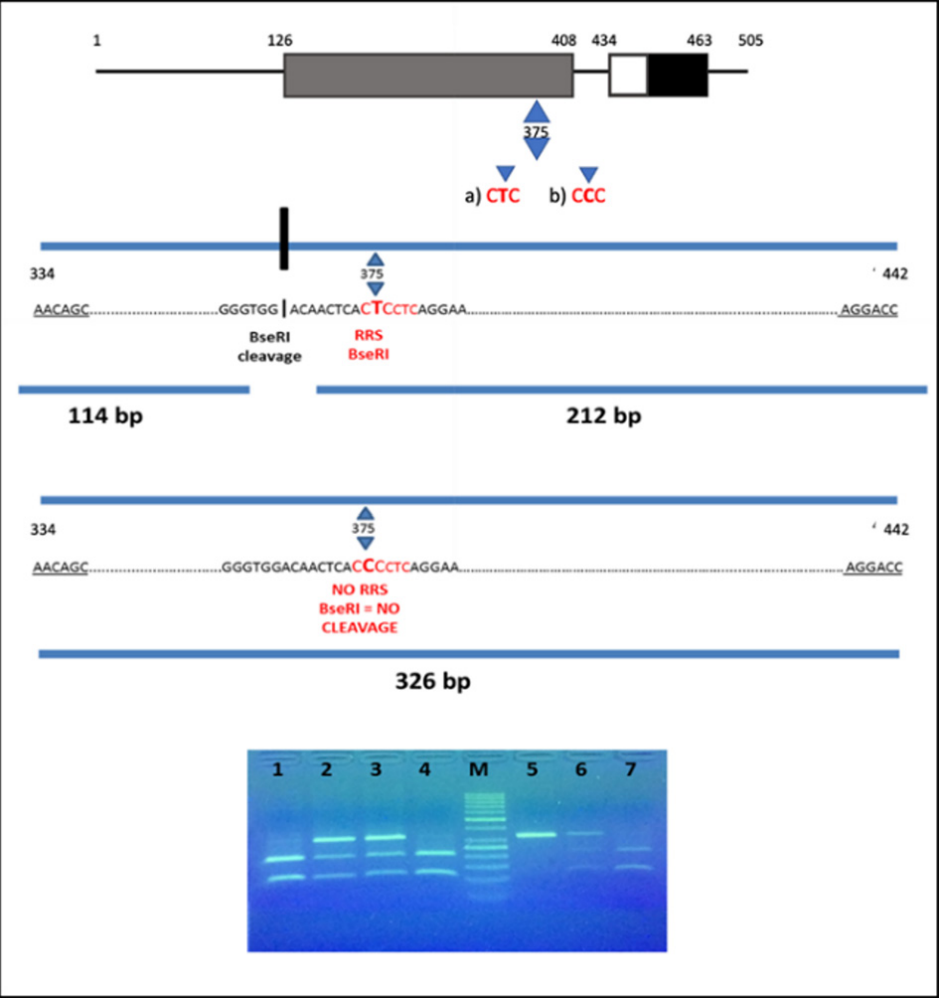
Identification of the E375G polymorphism (Glu^375^Gly variant) in CAMKK1, detected by BseRI restriction enzyme Top: schematic presentation of CAMKK1 domain structure. The conserved CaM-kinase domain is shown in gray (catalytic domain), white (autoinhibitory domain) and black (CaM-binding domains). The little triangles show the amino acid position of E375G polymorphism (CTC→CCC) in the kinase domain of CAMKK1. In (a) it is shown that the T allele generates a restriction recognition site (RRS) for BseRI. Its cleavage creates two fragments of 114 and 212 bp. In (b) it is shown that the C allele does not generate an RRS for BseRI, thus it can not cleave. The result is a fragment of 326 bp. Bottom: Electrophoresis on 3% agarose gel of BseRI restriction products: Lanes 1, 4 and 7, TT genotype (212 and 114 bp); s 2, 3 and 6, TC genotype (326, 212 and 114 bp); lane 5, CC genotype (326 bp); lane M, DNA ladder.

The polymorphic region of the CAMKK1 was amplified via PCR in a thermal cycler 2720 (Applied Biosystems) using two primers (synthesized by Eurofins) previously designed to amplify the region of 326 bp containing the single nucleotide polymorphism (SNP) of interest: forward primer: 5′-AACAGCACCGCCACCTTCATA-3′; reverse primer: 5′-GGTCCTTCTCATGTAATGGGAGC-3′. PCR was performed in a reaction volume of 25 µl using the DNA Dream Taq Polymerase (#EP0705, Thermo Fischer Scientific) using the following cycling conditions: denaturation, annealing, extension etc. After the amplification, the amplified PCR product of 326-bp fragment was detected using an UV transilluminator. PCR products were then digested using the restriction enzyme BseRI (#R0581L, NEB) in a total reaction volume of 25 µl. The rs7214723 polymorphic site is within the restriction site recognised by BseRI allowing the discrimination of the different genotypes. In presence of allele T, BseRI cuts the PCR products into two fragments of 114 and 212 bp; in presence of allele C, an intact fragment of 326 bp was obtained (Figure 2). Enzymatic digestions were carried out at 37°C for 1 h. The fragments were run on 3% agarose gel for approx. 50 min at 150 V and the bands were visualized on a UV transilluminator.

### Statistical analysis

As the subjects included in the present study were all cardiopathic patients, we referred to the 1000 Genomes SNP data (https://www.internationalgenome.org/1000-genomes-browsers/) [27], and we chose reference group: the European population.

Differences in genotypic and allelic frequencies between the two populations were examined by means of a Χ^2^ test. Hardy–Weinberg equilibrium (HWE) for CAMKK1 genotype distributions was tested by a goodness-of-fit Χ^2^ test.

The Χ^2^ test was also applied to analyze the differences in the distributions of age, sex, diabetes, hypertension, body mass index (BMI), previous history of neoplasia and dyslipidemia between CAD and NOCAD groups. A multiple comparison method was here applied using a simple Bonferroni correction technique (*P*=0.0071).

To estimate the association between the CAMKK1 polymorphism and the risk of CAD, odds ratio (OR) and 95% confidence intervals (95% CIs) were estimated using unconditional logistic regression, once controlled for age, sex, hypertension, diabetes, BMI, and previous history of neoplasia.

The STATA 15 software was used for all statistical analyses. The level of statistical significance was set at *P*<0.05.

## Results

### Characteristics of the cardiopathic population

The cardiopathic population was composed of subjects with different pathologies all requiring cardiac surgery. The most frequent condition was CAD, which was present in 150 subjects (CAD patients). Across CAD and NOCAD patients, 107 subjects presented AS. A little group, belonging to the NOCAD subpopulation, was composed of patients with mitral pathology.

CaMKK1 genotype distributions for rs7214723 polymorphism in the total cardiopathic population were not in HWE.

The characteristics of the total population study were analyzed considering the subdivision in CAD and NOCAD patients (Table 1). CAD and NOCAD resulted to have statistically different distributions in gender (*P*=0.000), incidence of diabetes (*P*=0.000) and dyslipidemia (*P*=0.000) (Table 1). This latter result further confirms the strong association between diabetes and coronary atherosclerotic disease. Conversely, the two subgroups did not differ significantly in age (*P*=0.033), hypertension (*P*=0.101), BMI (*P*=0.026) and previous history of neoplasia (*P*=0.558).

**Table 1:** Number and frequency distribution (in brackets) of selected characteristics of study subjects, divided in CAD and NOCAD groups

Variable	CAD	NOCAD	*P*-value
**Age (years)**			0.033
**<60**	19 (12.7%)	33 (22%)	
**≥60**	131 (87.3%)	117 (78%)	
**Gender**			0.000
**Male**	116 (77.3%)	76 (50.6%)	
**Female**	34 (22.7%)	74 (49.3%)	
**Hypertension**			0.101
**Yes**	120 (80.5%)	108 (72.5%)	
**No**	29 (19.5%)	41 (27.5%)	
**Diabetes**			0.000
**Yes**	55 (36.9%)	12 (8%)	
**No**	94 (63.1%)	137 (92%)	
**BMI**			0.026
**<25%**	44 (33.9%)	63 (47.3%)	
**≥25%**	86 (66.1%)	70 (52.7%)	
**Prev. neoplasia**			0.558
**Yes**	16 (10.8%)	13 (8.8%)	
**No**	133 (89.2°%)	136 (91.2%)	
**Dyslipidemia**			0.000
**Yes**	93 (62.4%)	54 (36.2%)	
**No**	56 (37.6%)	95 (63.8%)	

Abbreviation: Prev. neoplasia, previous history of neoplasia.

### Reference population

We retrieved the allele frequencies of the European population included in the 1000 Genomes Project for the rs7214723 polymorphism via Ensembl [27].

The European population includes 503 subjects from five different groups:

- CEU: population residing in Utah (U.S.A.) but with ancestors from Northern and Eastern Europe, made up 99 subjects
- FIN: Finnish population (Finland), made up 99 subjects;
- GBR: British population in England and Scotland (United Kingdom), made up 91 subjects;
- IBS: Iberian population (Spain), made up 107 subjects;
- TSI: Tuscan population (Italy) made up 107 subjects.

No other information about the distribution of sex, age, and other parameters are reported.

In Table 2, for each population group, the analysis of the genotype relating to the polymorphism rs7214723 is reported. The number and the frequencies of alleles and genotypes are also indicated.

**Table 2:** Analysis of the genotype relating to the polymorphism rs7214723 from databank 1000 Genomes

Population	Allele: frequency (count)		Genotype: frequency (count)		
**European (EUR)**	T: 0.557 (560)	C: 0.443 (446)	T|T: 0.318 (160)	C|C: 0.205 (103)	C|T: 0.477 (240)
-CEU	T: 0.520 (103)	C: 0.480 (95)	T|T: 0.273 (27)	C|C: 0.232 (23)	C|T: 0.495 (49)
-FIN	T: 0.631 (125)	C: 0.369 (73)	T|T: 0.404 (40)	C|C: 0.141 (14)	C|T: 0.455 (45)
-GBR	T: 0.560 (102)	C: 0.440 (80)	T|T: 0.286 (26)	C|C: 0.165 (15)	C|T: 0.549 (50)
-IBS	T: 0.509 (109)	C: 0.491 (105)	T|T: 0.290 (31)	C|C: 0.271 (29)	C|T: 0.439 (47)
-TSI	T: 0.565 (121)	C: 0.435 (93)	T|T: 0.336 (36)	C|C: 0.206 (22)	C|T: 0.458 (49)

It is reported: the gene frequencies both of alleles, on the right, and of genotypes on the left in the European population. Respectively, the total number of the allele and genotype is shown in brackets. Population description according to 1000 Genomes: CEU, Utah residents (CEPH) with Northern and Western European ancestry; FIN, Finnish in Finland; GBR, British in England and Scotland; IBS, Iberian population in Spain; TSI, Tuscan Italy.

### Genotypic frequencies

Genotypic frequencies were first compared between the total heart disease population (cardiopathic population) and the European reference population. Subsequently, several comparisons were made considering the groups of subjects with different heart diseases. (Figure 1).

Heterozygotes were more common in the cardiopathic than the reference group (Cardiopathic population TC = 64%; European TC = 48%;).

**Figure 3:**
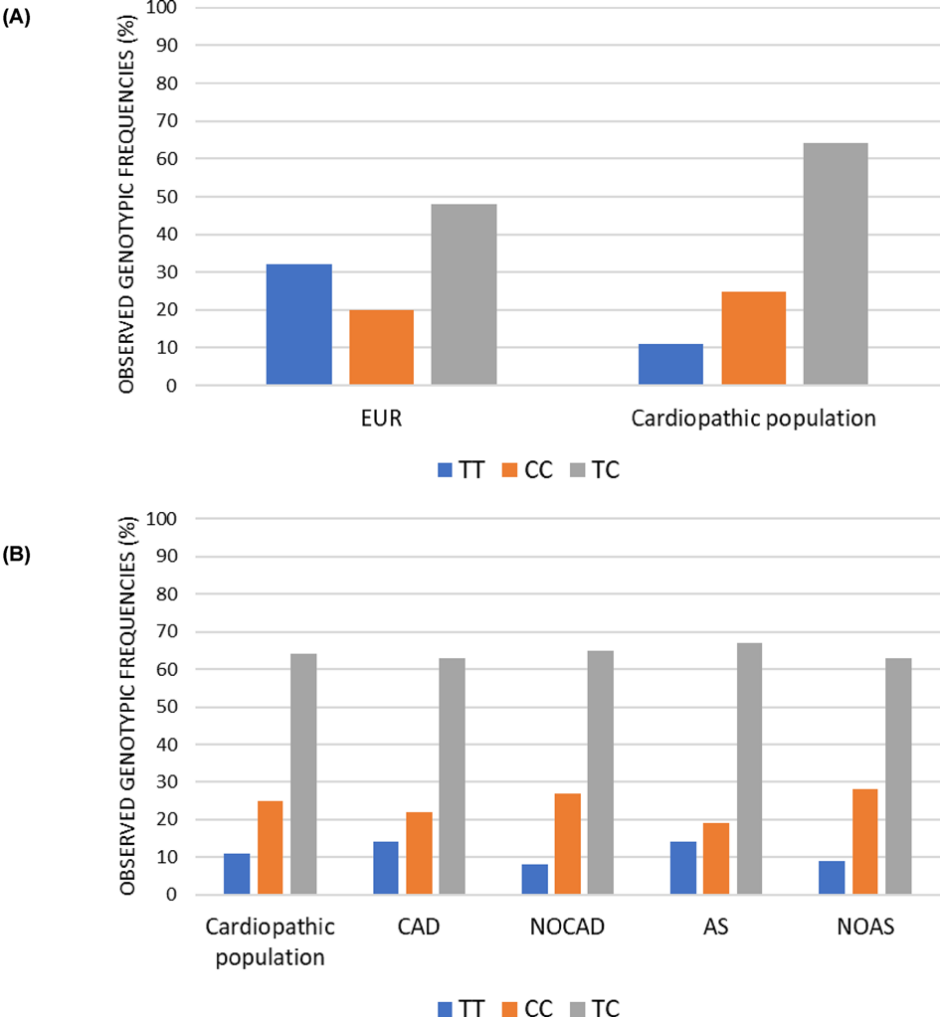
Observed genotypic frequencies (**A**) Histogram representation of the genotypic distribution frequencies relating to rs7214723 polymorphism of *CAMKK1* gene. All the frequencies are expressed in percentage. Cardiopathic population represents the total of 300 patients. The reference group used belongs to the European population (EUR). (**B**) Histogram representation of frequencies of the genotypic distribution relating to rs7214723 polymorphism of *CAMKK1* gene. All the frequencies are expressed in percentage. Focus on the pathology’s subdivision of CAD, NOCAD, AS and NOAS groups.

In the reference European population, the frequency of the ancestral TT genotype was higher than the CC genotype (TT = 32%; CC = 20%), while the opposite was observed in the total cardiopathic population (TT = 11%; CC = 25%) (Figure 3A).

A significant genotype difference distribution between the two groups was detected (*P*=0.000).

We then focused on patients and subdivided them in four groups on the basis of them presenting CAD and AS (Figure 3B). The CAD group was composed of 150 patients, and it was compared with the NOCAD group composed of 150 patients. Among these patients, there were 107 subjects with AS, of which 71 belonging to the NOCAD group and 36 to the CAD group, and 193 patients without AS, of which 79 belonged to the NOCAD group and 114 belonged to the CAD group. The Chi-square test performed between CAD and NOCAD groups and AS and no aortic stenosis (NOAS) groups did not reveal any statistical difference when the distribution of genotypes was considered (CAD vs NOCAD, *P*=0.225; AS vs NOAS, *P*=0.08).

### Allelic frequencies

In polymorphisms’ analysis, it is important to consider allelic frequencies. Inside a population, the minor allelic frequency (MAF) can be determined, and it is defined as the ratio between the frequency of the rarest variant and the most common variant of a specific SNP.

Allelic frequencies were first compared between the entire cardiopathic population sample and the European reference population. Subsequently, several comparisons were made between the groups of subjects with different heart diseases, as done for the genotypic analysis (Figure 1).

The polymorphism studied in the present paper, rs7214723, appears to be common in the population: the 1000 Genomes database reports a MAF index C = 0.3954/1980 and the T allele as the ancestral allele.

**Figure 4:**
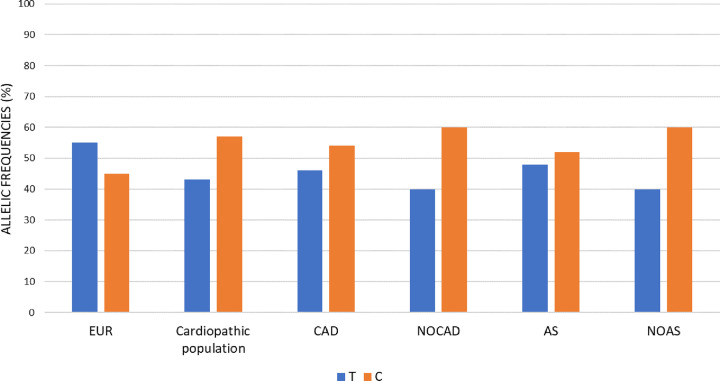
Allelic frequencies Histogram representation of allelic distribution frequencies relating to rs7214723 polymorphism of *CAMKK1* gene, both in reference European (EUR) group and the study groups of CAD, NOCAD, AS and NOAS. All the frequencies are expressed in percentage.

The allelic frequencies were therefore calculated both in the reference populations group (European) and in the CVDs cohort here are studied. As for the genotypic frequencies, alleles were present at different frequencies in the two groups. In the reference European population, the ancestral allele T was the predominant one, while in the total cardiopathic population, and in all the other subgroups (CAD; NOCAD; AS; NOAS) the C allele was the most frequent, especially in the non-CAD and non-AS subgroups (Figure 4).

### NOCAD stratification

Considering that AS patients comprised individuals with coronary condition patients and that AS has the same pathophysiological basis as coronary heart disease [2], we decided to further stratify the subgroup of non-coronary patients. The exclusion of the CAD patients from the AS group is clinically appropriate and can provides more power in testing for the phenotypic association with the SNP rs7214723.

Of the 150 non coronary patients, 79 had no AS (Figure 1).

**Figure 5:**
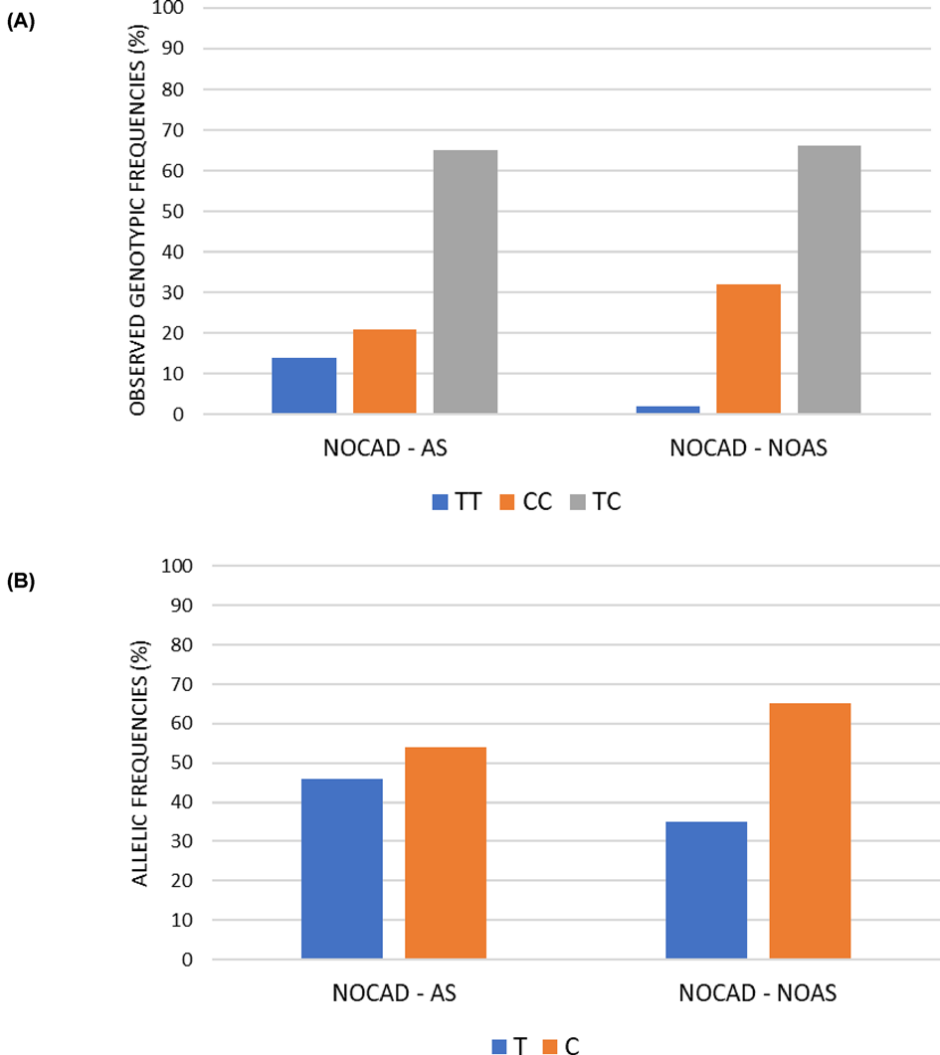
Focus on the pathology’s subdivision of AS and NOAS in NOCAD group (**A**) Histogram representation of frequencies of the genotypic distribution relating to rs7214723 polymorphism of *CAMKK1* gene. All the frequencies are expressed in percentage. (**B**) Histogram representation of allelic distribution frequencies relating to rs7214723 polymorphism of *CAMKK1* gene. All the frequencies are expressed in percentage.

The Chi-square test performed revealed statistical differences both for the genotypic (*P*=0.02) and allelic (*P*=0.05) distributions, the C allele and CC homozygotes more common in patients NOCAD with no AS compared with patients NOCAD with AS (Figure 5A,B).

### Logistic regression analysis

We then tested a possible direct association between polymorphism rs7214723 in CAMKK1 and the risk of developing a specific CVD. Two models were estimated: the first concerned the risk of developing CAD, while the second one focused on AS.

Logistic regression analysis was applied to analyze simultaneously the influence of independent variables (genotype, sex, age, diabetes, hypertension, BMI and previous history of neoplasia) on the risk of developing CAD and AS (Tables 3 and 4, respectively). In both tables, the frequencies of the genotypes for rs7214723 polymorphism for CAD/NOCAD and AS/NOAS are shown.

**Table 3:** Analysis of association between CAMKK1 rs7214723 and risk of CAD, through an unconditional logistic regression analysis adjusted for sex, age, diabetes, hypertension, BMI, previous history of neoplasia

rs7214723	CAD no.(%)	NOCAD no. (%)	OR (95% CI)	Robust Std. Err.	*P*> |Z|
Additive model					
**Genotype**					
TT	21 (14%)	12 (8%)	1.000 (reference)		
TC	95 (63.4%)	98 (65.4%)	0.496 (0.199–1.235)	0.230	0.132
CC	34 (22.6%)	40 (26.6%)	0.516 (0.185–1.437)	0.269	0.206
**Age**					
<60	19 (12.7%)	33 (22%)	0.468 (0.225–0.974)	0.175	0.043
≥60	131 (87.3%)	117 (78%)	1.000 (reference)		
**Sex**					
M	116 (77.3%)	76 (50.6%)	4.018 (2.210–7.305)	1.225	0.000
F	34 (22.7%)	74 (49.3%)	1.000 (reference)		
**Hypertension**					
YES	120 (80.5%)	108 (72.5%)	1.077 (0.566–2.049)	0.353	0.820
NO	29 (19.5%)	41 (27.5%)	1.000 (reference)		
**Diabetes**					
YES	55 (36.9%)	12 (8%)	5.540 (2.524–12.160)	2.222	0.000
NO	94 (63.1%)	137 (92%)	1.000 (reference)		
**Pre. neoplasia**					
YES	16 (10.8%)	13 (8.8%)	0.848 (0.299–2.397)	0.449	0.756
NO	133 (89.2%)	136 (91.2%)	1.000 (reference)		
**BMI**					
≥25%	86 (66.1%)	70 (52.7%)	1.220 (0.684–2.177)	0.360	0.499
<25%	44 (33.9%)	63 (47.3%)	1.000 (reference)		
Recessive model					
**Genotype**					
TT+TC	116 (77.3%)	110 (73.4%)	1.000 (reference)		
CC	34 (22.7%)	40 (26.6%)	0.949 (0.504–1.786)	0.306	0.872
**Age**					
<60	19 (12.7%)	33 (22%)	0.445 (0.215–0.922)	0.165	0.029
≥60	131 (87.3%)	117 (78%)	1.000 (reference)		
**Sex**					
M	116 (77.3%)	76 (50.6%)	3.732 (2.074–6.717)	1.119	0.000
F	34 (22.7%)	74 (49.3%)	1.000 (reference)		
**Hypertension**					
YES	120 (80.5%)	108 (72.5%)	1.069 (0.568–2.013)	0.345	0.835
NO	29 (19.5%)	41 (27.5%)	1.000 (reference)		
**Diabetes**					
YES	55 (36.9%)	12 (8%)	5.762 (2.640–12.573)	2.293	0.000
NO	94 (63.1%)	137 (92%)	1.000 (reference)		
**Pre. neoplasia**					
YES	16 (10.8%)	13 (8.8%)	0.843 (0.301–2.359)	0.442	0.746
NO	133 (89.2%)	136 (91.2%)	1.000 (reference)		
**BMI**					
≥25%	86 (66.1%)	70 (52.7%)	1.240 (0.698–2.200)	0.362	0.462
<25%	44 (33.9%)	63 (47.3%)	1.000 (reference)		
Dominant model					
**Genotype**					
TT	21 (14%)	12 (8%)	0.501 (0.204–1.231)		
CC+TC	129 (86%)	138 (92%)	1.000 (reference)	0.204	0.132
**Age**					
<60	19 (12.7%)	33 (22%)	0.469 (0.226–0.974)	0.174	0.042
≥60	131 (87.3%)	117 (78%)	1.000 (reference)		
**Sex**					
M	116 (77.3%)	76 (50.6%)	4.019 (2.212–7.305)	1.225	0.000
F	34 (22.7%)	74 (49.3%)	1.000 (reference)		
**Hypertension**					
YES	120 (80.5%)	108 (72.5%)	1.077 (0.566–2.048)	0.353	0.821
NO	29 (19.5%)	41 (27.5%)	1.000 (reference)		
**Diabetes**					
YES	55 (36.9%)	12 (8%)	5.528 (2.519–12.133)	2.217	0.000
NO	94 (63.1%)	137 (92%)	1.000 (reference)		
**Pre. neoplasia**					
YES	16 (10.8%)	13 (8.8%)	0.852 (0.302–2.396)	0.449	0.761
NO	133 (89.2%)	136 (91.2%)	1.000 (reference)		
**BMI**					
≥25%	86 (66.1%)	70 (52.7%)	1.220 (0.684–2.176)	0.360	0.499
<25%	44 (33.9%)	63 (47.3%)	1.000 (reference)		

Prev. neoplasia, previous history of neoplasia; *P*> |Z|, *P*-value from Z score; Robust Std. Err., robust standard error.

Additive model

Log pseudolikelihood = −151.48372

Prob > χ^2^ = 0.0000

Recessive model

Log pseudolikelihood = −152.62122

Prob > χ^2^ = 0.0000

Dominant model

Log pseudolikelihood = −151.49063

Prob > χ^2^ = 0.0000

**Table 4:** Analysis of association between CAMKK1 rs7214723 and risk of AS, through an unconditional logistic regression analysis adjusted for sex, age, diabetes, hypertension, BMI and previous history of neoplasia

rs7214723	AS no. (%)	NOAS no. (%)	OR (95% CI)	Robust Std. Err.	P> |Z|
Additive model					
**Genotype**					
TT	16 (15%)	17 (8.8%)	1.000 (reference)		
TC	71 (66.4%)	122 (63.2%)	0.668 (0.303–1.475)	0.270	0.319
CC	20 (18.6%)	54 (28%)	0.466 (0.185–1.177	0.220	0.107
**Age**					
<60	10 (9.3%)	42 (21.8%)	0.518 (0.223–1.204)	0.222	0.127
≥60	97 (90.7%)	151 (78.2%)	1.000 (reference)		
**Sex**					
M	56 (52.3%)	136 (70.5%)	0.445 (0.256–0.774)	0.125	0.004
F	51 (47.7%)	57 (29.5%)	1.000 (reference)		
**Hypertension**					
YES	88 (82.2%)	140 (73.3%)	1.297 (0.657–2.559)	0.449	0.453
NO	19 17.8%)	51 (26.7%)	1.000 (reference)		
**Diabetes**					
YES	23 (21.5%)	44 (23%)	0.789 (0.411–1.513)	0.262	0.476
NO	84 (78.5%)	147 (77%)	1.000 (reference)		
**Pre. neoplasia**					
YES	15 (14%)	14 (7.3%)	1.780 (0.704–4.499)	0.842	0.223
NO	92 (86%)	177 (92.7%)	1.000 (reference)		
**BMI**					
≥25%	52 (53.6%)	112 (62.2%)	1.183 (0.682–2.053)	0.332	0.549
<25%	45 (46.4%)	68 (37.8%)	1.000 (reference)		
Recessive model					
**Genotype**					
TT+TC	87 (81.3%)	139 (72%)	1.000 (reference)		
CC	20 (18.7%)	54 (28%)	0.657 (0.349–1.236)	0.211	0.193
**Age**					
<60	10 (9.3%)	42 (21.8%)	0.500 (0.215–1.159)	0.214	0.106
≥60	97 (90.7%)	151 (78.2%)	1.000 (reference)		
**Sex**					
M	56 (52.3%)	136 (70.5%)	0.433 (0.250–0.752)	0.121	0.003
F	51 (47.7%)	57 (29.5%)	1.000 (reference)		
**Hypertension**					
YES	88 (82.2%)	140 (73.3%)	1.299 (0.655–2.575)	0.453	0.453
NO	19 17.8%)	51 (26.7%)	1.000 (reference)		
**Diabetes**					
YES	23 (21.5%)	44 (23%)	0.823 (0.435–1.557)	0.267	0.550
NO	84 (78.5%)	147 (77%)	1.000 (reference)		
**Pre. neoplasia**					
YES	15 (14%)	14 (7.3%)	1.797 (0.705–4.580)	0.857	0.219
NO	92 (86%)	177 (92.7%)	1.000 (reference)		
**BMI**					
≥25%	52 (53.6%)	112 (62.2%)	1.193 (0.687–2.069)	0.335	0.530
<25%	45 (46.4%)	68 (37.8%)	1.000 (reference)		
Dominant model					
**Genotype**					
TT	16 (15%)	17 (8.8%)	0.613 (0.282–1.334)	0.243	0.218
CC+TC	91 (85%)	176 (91.1%)	1.000 (reference)		
**Age**					
< 60	10 (9.3%)	42 (21.8%)	0.509 (0.219–1.184)	0.218	0.117
≥ 60	97 (90.7%)	151 (78.2%)	1.000 (reference)		
**Sex**					
M	56 (52.3%)	136 (70.5%)	0.445 (0.258–0.769)	0.124	0.004
F	51 (47.7%)	57 (29.5%)	1.000 (reference)		
**Hypertension**					
YES	88 (82.2%)	140 (73.3%)	1.291 (0.657–2.535)	0.444	0.458
NO	19 17.8%)	51 (26.7%)	1.000 (reference)		
**Diabetes**					
YES	23 (21.5%)	44 (23%)	0.803 (0.421–1.533)	0.264	0.508
NO	84 (78.5%)	147 (77%)	1.000 (reference)		
**Pre. neoplasia**					
YES	15 (14%)	14 (7.3%)	1.718 (0.694–4.253)	0.794	0.242
NO	92 (86%)	177 (92.7%)	1.000 (reference)		
**BMI**					
≥25%	52 (53.6%)	112 (62.2%)	1.186 (0.684–2.056)	0.332	0.541
<25%	45 (46.4%)	68 (37.8%)	1.000 (reference)		

Abbreviations: Prev. neoplasia, previous history of neoplasia; *P*> |Z|, *P*-value from Z score; Robust Std. Err., robust standard error.

Additive model

Log pseudolikelihood = −162.35768

Prob > χ^2^ = 0.0177

Recessive model

Log pseudolikelihood = −162.83012

Prob > χ^2^ = 0.0112

Dominant model

Log pseudolikelihood = −162.95108

Prob > χ^2^ = 0.0171

Three different genetic models were considered in our analysis: additive model (TT; TC; CC), dominant model (CC+TC versus TT), and recessive model (CC versus TC+TT), considering C as the minor allele (C) in accordance with the frequency reported in the 1000 Genomes Database [27].

Not a significant association between the rs7214723 polymorphism and the risk of CAD was reported independently of the model taken in consideration (additive, recessive, and dominant; Table 3).

On the contrary, in male participants the CAMKK1 rs7214723 polymorphism resulted positively associated with the increased risk of CAD in all the three models, additive: OR = 4.018 (2.210–7.305), *P*=0.000); recessive: OR = 3.73 (2.074–6.717), *P*=0.000; dominant: OR = 4.019 (2.212–7.305), *P*=0.000).

Similarly, patients under 60 years and non-diabetic patients appear to have a lower risk to develop CAD irrespective of the model taken in consideration, but no association was found for hypertension, BMI, and previous history of neoplasia (Table 3).

When the rs7214723 polymorphism was tested for association with the risk of AS, male participants showed positive association. On the contrary, none of the other variables provided a significant result (Table 4).

## Discussion

Over the last few years, genetic variants have raised interest for their relevance in different contexts. Polymorphisms have in fact been shown to have important roles as biomarkers for the prognosis and diagnosis of diseases, health prevention, epidemiology, and pharmacology [28].

The identification and study of genetic variants involved in multifactorial diseases is particularly important given the complexity of their underlying genetic architecture.

Within this context, CVDs are extremely important to investigate as represent one of the main causes of death in industrialized countries. For example, the search for specific genetic and epigenetic biomarkers that can cooperate with the canonical markers (e.g. troponin) in the diagnosis, prognosis, and risk prediction of CVDs might significantly contribute in improving the health status of industrialized societies [29].

In this context, it is really important to consider also the epidemiology of the CVDs and the role played by non-genetic factors as the environment, climate change, historical heritage and different lifestyle habits.

In a previous review, we discussed the several SNPs associated with CVDs in genes involved in the calcium/calmodulin pathway [14–21]. In particular, this study focused on some downstream genes to calmodulin, the first calcium sensor and signal transducer. In fact, this pathway is involved in the reception and transduction of the calcium signaling, that plays a key role in the heart being involved in different processes. This review highlights the high number of studies regarding nitric oxide synthase gene (*NOS*), especially the endothelial isoform (eNOS). Different studies have suggested the important role of eNOS in CVDs and how genetic variants might affect its activity and increase the higher risk to develop CVDs. In fact, the principal role of eNOS is the synthesis of nitric oxide, that plays a crucial role in improving vascular density and maintaining cardiac performance [30,31]. Genetic variants in eNOS have been associated with low nitric oxide concentrations and vascular density [32–35].

Among these genes, the calcium/calmodulin kinase family proteins represent a group of genes particularly associated with CVDs [24,36,37]. Among them, CAMK2 is the most studied in the contest of the physiology of the heart [38–40]. CAMK2 has a key role in the excitation contraction coupling [41–43] because of its phosphorylation activity on several Ca^2+^ handling proteins, including *sarcoplasmic reticulum* (SR) Ca^2+^ release channels or ryanodine receptors (RyRs) [44], phospholamban (PLB) [45], and L-type Ca^2+^ channels [41]. Only a few genetic variants within this gene have been tested for their association with disease metabolism and CAD.

In the present study we focused on another member of the calcium/calmodulin kinase family, CAMKK1, in particular testing for the potential association between the SNP rs7214723 and a higher risk to develop CVDs. CAMKK1 is a calcium calmodulin dependent kinase kinase 1, a protein of the calcium calmodulin pathway.

The C to T variants lead to an amino acid change inside the kinase domain of CAMKK1 (E375G) which might create a conformational change and decrease it. This might contribute to modulate the calcium signaling pathway and thus increase the susceptibility to CVDs.

In the present study, we have analyzed the distribution of the genetic frequencies, related to rs7214723, in a cardiopathic population composed of 300 subjects and in a European population taken from the 1000 Genomes database [27].

The data obtained in the different genotypic distribution among European and cardiopathic study population resulted statistically significant: first, it was observed an increase of the homozygous frequency (TC) in a cardiopathic study group (Cardiopathic population TC = 64%; European TC = 48%). Moreover, it was possible to highlight the opposite trend of the homozygous genotype (TT and CC), between the two groups.

In the reference population groups, the frequency of TT was higher than that CC. On the other hand, in the population study, the trend was exactly the opposite: the frequency of CC was higher than that TT.

The same trend was observed also for the subgroups (CAD, NOCAD, AS, NOAS) (Figure 3 and Supplementary Table S1). Specifically, in the NOCAD and NOAS, the percentage of CC was the higher (NOCAD: CC = 27%; NOAS: CC = 28%, CAD: CC = 22%; AS: CC = 19%).

Considering that the study population group was a selected and specific group of cardiopathic subjects requiring cardiac surgery, it was interesting to observe the enrichment for the non-ancestral genotype CC, particularly in subjects with NOCAD and NOAS.

The increase in a population specifically selected might suggest a higher risk for the subjects with the CC genotype to develop CVD. The enrichment specifically observed in the NOCAD and NOAS subgroups might suggest a major difficulty in bearing the specific damage caused to the tissue by no CAD (as valve diseases) or AS.

In view of the interesting data of the NOCAD and NOAS subgroups, the no coronary subgroup was further stratified to exclude from the AS and NOAS subgroups the patients with CAD, since these two pathologies have a similar pathophysiological basis. This stratification highlighted that no-coronary patients with no-AS have an higher enrichment of the C allele compared with no-coronary patients with AS (NOCAD/NOAS C = 65%, NOCAD/AS C = 54%).

The logistic regression analysis adjusted for sex, age, diabetes, hypertension, BMI, and previous history of neoplasia showed no association between polymorphism rs7214723 in CAMMK1 and the risk to develop CAD or AS was not found.

However, for male participants, CAMKK1 rs7214723 polymorphism showed a positive correlation with the increased risk of both CAD and AS.

The significance of the logistic regression analysis performed in the subdivision CAD/NOCAD held true only for diabetics and patients older than 60 years a higher risk to develop CAD. There was no significant association in the analysis of hypertension, BMI, and previous history of neoplasia.

The results obtained from the logistic regression analysis of AS/NOAS showed no significant association with the analysis of age, diabetes, hypertension, BMI, and previous history of neoplasia.

In summary, the results obtained in the present study suggest the need to better understanding the molecular and biological role of the *CAMMK1* gene in CVDs. The amino acid change (E375G) within the kinase domain of CAMKK1 might have a decisive role in the kinase activity of the protein influencing the downstream pathways of calcium signaling. This event might contribute to develop heart problems, increasing the predisposition to this kind of disease.

Several limitations exist in the present study. First, the small sample size of the population could have limited the statistical power to detect additional significant associations for each of the pathological types. Second, internal controls may need to be undertaken. Furthermore, considering that CVDs are multifactorial diseases, the comparison of the genetic data of the study population with the data relating to the database of 1000 genomes did not allow the evaluation of concomitant genetic and environmental factors in cases and controls. Considering this, it will be important in the future to perform gene association studies in order to investigate whether the combination of all polymorphisms of interest in the calcium calmodulin pathway, each with small effect, it could affect the development of the disease, more than a single variant itself. Further prospective population-based studies and biochemical experiments are needed to verify the conclusion.

Some evidence indicates that in addition to a possible role of polymorphic variants in the CAMKK1 protein, epigenetic modifications associated with the modulating activity of this protein could have an impact on cardiovascular risk. In this context, future studies will also be aimed at investigating the impact on the epigenome of both the different genotypes identified and of different concentrations of the protein.

## Supplementary Material

Supplementary Table S1

## Data Availability

The data that support the findings of the present study are available from the corresponding author upon reasonable request.

## Abbreviations

AS: aortic stenosis
BMI: body mass index
CAD: coronary artery disease
CAMK: calcium/calmodulin- dependent kinase
CAMKK1: calcium/calmodulin-dependent kinase kinase 1
CVD: cardiovascular disease
eNOS: endothelial isoform of nitric oxide synthase
HWE: Hardy–Weinberg equilibrium
LV: leaflet valve
MAF: minor allelic frequency
MSC: mesenchimal stem cell
NOAS: no aortic stenosis
PPP3C: protein phosphatase 3 catalytic subunit
SNP: single nucleotide polymorphism
