# Supplementary material for: Polymorphism rs7214723 in CAMKK1: a new genetic variant associated with cardiovascular diseases
Source: Biosci Rep. 2021 Jul 6;41(7):BSR20210326. doi: 10.1042/BSR20210326 (PMC8264181; doi:10.1042/BSR20210326)
Supplement: Supplementary Table S1 [file BSR-2021-0326_supp.pdf]

SUPPLEMENTARY MATERIAL

Polymorphism rs7214723 in CAMKK1: a new genetic variant associated with cardiovascular diseases.

Sofia Beghi[1], Francesca Cavaliere[2], Matteo Manfredini[1], Sandro Ferrarese[3], Claudio Corazzari[3], Cesare Beghi[3], Annamaria Buschini[1]*

[1] University of Parma; Department of Chemistry, Life Sciences, and Environmental Sustainability; Parco Area delle Scienze 11A; 43124 Parma; Italy
[2] University of Parma; Department of Food and Drug; Parco Area delle Scienze 27A; 43124 Parma; Italy
[3] Department of Cardiac Surgery, Circolo Hospital, University of Insubria, Varese, Italy.

Table S1 - Number of subjects, allelic and genotypic frequency distributions of the populations under study. CAD (coronary artery disease); NOCAD (no coronary artery disease); AS (aortic stenosis); NOAS (no aortic stenosis).

Population (n. subjects)	Allelic frequency (count)	Genotypic frequency (count)	p value (genotypic frequency)
European population (503)	T: 55% (560)<br>C: 45% (446)	TT: 32% (160)<br>CC: 20% (103)<br>CT: 48% (240)	EUR vs Cardiopathic population<br>p=0.000
Cardiopathic population (300)	T: 43% (259)<br>C: 57% (341)	TT: 11% (33)<br>CC: 25% (74)<br>CT: 64% (193)	
CAD (150)	T: 46% (137)<br>C: 54% (163)	TT: 14% (21)<br>CC: 22% (34)<br>CT: 63% (95)	CAD vs NOCAD<br>p= 0.225
NOCAD (150)	T: 40% (122)<br>C: 60% (178)	TT: 8% (12)<br>CC: 27% (40)<br>CT: 65% (98)	
AS (107)	T: 49% (103)<br>C: 51% (111)	TT: 14% (16)<br>CC: 19% (20)<br>CT: 67% (71)	AS vs. NOAS<br>p=0.08
NOAS (193)	T: 40% (156)<br>C: 60% (230)	TT: 9% (17)<br>CC: 28% (54)<br>CT: 63% (122)	

Table S1 - Number of subjects, allelic and genotypic frequency distributions of the populations under study. CAD (coronary artery disease); NOCAD (no coronary artery disease); AS (aortic stenosis); NOAS (no aortic stenosis).
